# Determinants of Socioeconomic Inequalities in Well-Being in Canada: Evidence From the Nova Scotia Quality of Life Survey

**DOI:** 10.34172/ijhpm.8643

**Published:** 2025-01-22

**Authors:** Daniel Keays, Mohammad Hajizadeh

**Affiliations:** ^1^Sobey School of Business, Saint Mary’s University, Halifax, NS, Canada.; ^2^School of Health Administration, Dalhousie University, Halifax, NS, Canada.

**Keywords:** Socioeconomic Inequalities, Canadian Index of Wellbeing, Concentration Index, Nova Scotia, Canada

## Abstract

There are relatively few studies that have measured and explained socioeconomic inequalities in the well-being of populations. Using unique information available in the 2019 Nova Scotia Quality of Life Survey (NSQLS, n=9388), this study provides analysis of the determinants of socioeconomic inequalities in well-being of adults aged 18 and above in Nova Scotia, Canada. The population’s well-being was measured using the Canadian Index of Wellbeing (CIW), which encompasses quality of life across eight domains. The Concentration index (C) approach was utilized to quantify and identify factors explaining socioeconomic inequality in well-being. A positive value of the C (0.0294; 95% confidence interval: 0.0267 to 0.0321) indicated pro-rich inequality in well-being among Nova Scotian residents. Results of the decomposition analysis indicated that the concentration of favorable mental health, education levels, and income among high socioeconomic status (SES) groups accounted for over 86% of the observed socioeconomic inequality in the population’s well-being. Our findings demonstrated that inequalities in mental health, education, and income are significant obstacles to reducing inequality in well-being in Nova Scotia, Canada. Thus, policies aimed at alleviating inequalities in these factors may help to reduce socioeconomic inequality in well-being in Nova Scotia, Canada.

## Introduction

 Improving the overall well-being of a society is a significant public policy objective across nations globally^1-3^ including within Canada.^4-6^ In fact, developing strategies and policies to enhance the well-being of residents is a critical objective for nations globally for several reasons. Notably, a healthy or improved state of well-being enables individuals to pursue and realize their personal ambitions and uphold the values they possess.^7^ In other words, improving the well-being for individuals brings much personal fulfillment, and thus raises the overall livelihood for those individuals.

 Policies aimed specifically at increasing well-being levels can relate to a wide range of issues and domains. Moreover, determining the most effective approaches to enhance well-being through government policies can present a complex task for public policy-makers. That is not to mention the various stakeholders who are interrelated, and connected, to certain political parties and political agendas, which, in turn, also contribute to the complexity of certain policy initiatives.^8^

 Although the well-being of society is a primary concern, the foremost objective of governments across the globe is not simply to enhance well-being but to promote equitable distribution of it amongst all citizens.^4,9,10^ In other words, variation in well-being among different subgroups of the population is one of the specific concerns for governments. Addressing these inequalities is, and continues to be, a major topic of interest for policy decision-making globally.

 Inequalities in well-being within populations can be driven by several different factors, that, in many instances, are related to one another to a certain extent. Previous research related to well-being literature highlighted macro and micro level of factors associated with the inequalities in well-being measures. Specifically, population well-being can be classified into individual level indicators (micro level, eg, community vitality, income and wealth, and education, and time use) as well as nation-wide, or national level (macro level, eg, environmental quality, national security, and good governance) indicators.^11,12^ At each level, whether micro or macro, the impact on well-being varies according to specific contexts at large. For instance, a country’s level of development, as reflected in its economic prosperity, can significantly influence how various factors affect the well-being of its citizens.^13^

 Previous studies have identified socioeconomic status (SES), encompassing factors such as education and income, as a key micro-level determinant that significantly influences inequalities in well-being outcomes globally.^9,11,14,15^ The current literature investigating the relationship between income and well-being^16-20^ has highlighted both the absolute and relative income effects, and how they have had unique ways of effecting the well-being of populations.^21,22^ With that being said, this current study offers a fresh perspective on the determinants of income inequality in well-being by utilizing the Canadian Index of Wellbeing (CIW), which employs a multidimensional framework to assess various aspects of quality of life, to measure and explain socioeconomic inequality in well-being in Nova Scotia, Canada.

 Current research on inequalities in well-being within Canada remains limited. Although various studies have explored different aspects of well-being,^23-25^ there is a noticeable gap in the literature specifically focused on measuring and explaining socioeconomic inequalities in well-being. For instance, a study by Dilmaghani investigates the relationship between religion and subjective well-being (SWB) in Quebec, Canada.^26^ The latter study, however, does not take further look at determinants or inequalities in the well-being for this province. We aim to address this research gap by investigating socioeconomic inequalities in well-being within Nova Scotia, which is situated in Canada’s “Atlantic Provinces” at the most eastern part of the country. This province was chosen for our study given the availability of the unique 2019 Nova Scotia Quality of Life Survey (NSQLS) dataset, and it has a population of approximately one million.^27^ Utilizing the 2019 NSQLS and employing the Concentration index (C) approach, this study quantifies income-related inequality in well-being in Nova Scotia. Additionally, we decompose this income-related inequality to identify factors contributing to the observed inequality in well-being within the province.

## Methods

###  Data

 Data were derived from a confidential master file of the NSQLS, a large survey conducted in 2019 by the Engage Nova Scotia organization. The survey was designed to provide an unbiased and deep understanding of what individuals and communities are experiencing across Nova Scotia. As a large cross-sectional survey, it collected data from the province residents related to economic and social aspects of their lives. The survey phase was launched in the spring of 2019 with two principal approaches to encourage residents of the province to participate. The approaches used to encourage residents who were 16 years of age or older to participate in the survey were: (1) a personalised letter to approximately 80 000 randomly selected households from across 10 relatively distinct regions covering the entire province; and (2) a targeted outreach to specific populations who might not typically have the same opportunity to complete a questionnaire using traditional survey approaches (ie, lower income residents; younger residents; immigrants/refugees; people living with disabilities; older adults). The random selection of households included an oversampling of the more rural regions of the province to ensure adequate representation of residents living in smaller communities and outlying areas. To help ensure a broader age range of respondents, the household member 16 years of age or older with a birthday closest to June 1st was asked to complete the survey. Approximately 14 000 questionnaires were initially submitted by Nova Scotians, corresponding to a response rate of about 16% based on the total number of eligible participants (80 000). Of these, 12 826 questionnaires were assessed as usable, resulting in an approximate completion rate of 92% among those who started the survey. Most surveys were completed online (88.6%; n = 11 363) with the remaining surveys either completed using a paper version that was either requested by residents (6.0%; n = 764) or used with targeted groups as part of the outreach approach (5.4%; n = 699).^28^ Some aspects of the survey include questions about education, income, housing, employment, physical and mental health, and more.^28^ The selected households received requests to participate in the 230-question survey, and the survey took approximately 30 minutes to complete. Invitations to fill out the survey, with a personalized 5-digit access code, was sent to participate through mail. Additional targeted outreach was completed, through a variety of means, to groups that might not otherwise have their voices heard.^29^ The survey was sent to one in five households in Nova Scotia. Further, to ensure that the results from the survey were representative of the residents of Nova Scotia, the data provided by the total respondents were weighted by sex, age grouping, and region to match the Census profile for 2019 of those individuals aged 16 and older.^28^ Through the deployment of various recruitment strategies tailored to the survey’s nature, and informed by previous experience with general population surveys, the survey successfully recruited a total of 12 871 participants. After excluding participants younger than 18 years old and observations with missing information on the variables included in our analysis, the final sample size for our study comprised 9388 respondents.

###  Variables

*Outcome variable*: The main outcome variable in the study is the measure of individual well-being. The concept of “well-being” encompasses a diverse array of measurement approaches, each unique and distinct. The methods for collecting well-being data and the criteria used to evaluate it can vary widely across countries.^30^ In Canada, individual well-being has been assessed using both subjective and objective measures across various research fields.^31-34^ SWB emphasizes personal perceptions and feelings, highlighting the importance of individual experiences. These types of questions, incorporating SWB measures, are also embedded within the broader CIW framework.^34,35^ In our study, we utilized the CIW to assess the well-being of participants in the NSQLS. The CIW is a composite index of eight interconnected domains that measures trends in the well-being of Canadians over time. Well-being is defined as “the presence of the highest possible quality of life in its full breadth of expression focused on but not necessarily exclusive to: good living standards, robust health, a sustainable environment, vital communities, an educated populace, balanced time use, high levels of democratic participation, and access to and participation in leisure and culture.”^36^ Respondents were asked to answer, and rank, several questions related to the eight CIW components on a scale of 1 to 7. The value of 1 reflected that the respondent was feeling “extremely dissatisfied,” while a ranking of 7 meant the respondent was “extremely satisfied.” The overall CIW score is computed by averaging the scores from each domain and then combining them to generate the total CIW score. In other words, for a given year, the mean composite scores of the eight domains are summed and then divided by eight, which generates an overall measure of well-being for Canadians in that given time frame. One of the goals of the CIW is to identify and examine interconnections among the many factors influencing the well-being of Canadians.^36^ This approach brings a novel and distinctive perspective to understanding the well-being of a population.^37,38^

*Socioeconomic and other control variables*: Income, which is commonly used as a measure of SES, was employed as an indicator of SES in the estimation of the C for well-being. As household income was collected as a categorical variable in the survey, we assigned the mid-point value of each category to represent household income. We employed formulas outlined by Parker and Fenwick,^39^ which are derived from the principles of Pareto’s law of income distribution, to calculate the mid-point for the upper income category. We then computed equivalized household income by dividing the household income by the square root of the household size. Based on the previous literature in the related topic,^40-42^ a variety of socioeconomic and demographic variables available in the survey data set (ethnicity, income, mental health status, education, employment, and region) were used as explanatory variables. We used income measured at the household level because it has been shown, through prior studies, to be a better measure of SES than individual level income.^43,44^ Table 1 reports the definitions of all the variables used in the study.

**Table 1:** Definition of Variables Used in the Study

**Variable Name**	**Description**
**Outcome variable**	
Well-being	Eight quality of life categories or domains including: community vitality, democratic engagement, education, environment, healthy populations, leisure and culture, living standards, and time use. Ranked from 1 (extremely dissatisfied) to 7 (extremely satisfied)
**Independent variables**	
Sociodemographic variables	
Age	Age in years (18+)
Male	1 = if male, 0 otherwise
Marital status	
Single, never married	1 = if the individual is single and never married, 0 otherwise
Married or living common-law	1 = if the individual is married or living common law, 0 otherwise
Separated, divorced, or widowed	1 = if the individual is separated, divorced, or widowed, 0 otherwise
Ethnicity	
Minority	1 = if the respondent falls into ethnic minority group, 0 otherwise
Non-minority	1 = if the respondent does not fall into ethnic minority group, 0 otherwise
Socioeconomic status	
Equivalized household income	Total equivalized household income before taxes from all sources for year
Education	
Elementary or high school	1= if the highest level of education completed is elementary or high school, 0 otherwise
Post-secondary, trade	1 = if the highest level of education completed is post- secondary, trade, or apprenticeship, 0 otherwise
College diploma	1 = if the highest level of education completed is college diploma, 0 otherwise
University degree	1 = if the highest level of education completed is university degree, 0 otherwise
Graduate degree	1 = if the highest level of education completed is graduate degree, 0 otherwise
Employment status	
Employed	1 = if the respondent is employed, 0 otherwise
Unemployed	1 = if the respondent is unemployed, 0 otherwise
Retired	1 = if the respondent is retired, 0 otherwise
Other employment	1 = if the respondent has other employment status (student, housework, etc), 0 otherwise
Mental health	
Excellent	1= if the respondent reported excellent mental health status, 0 otherwise
Very good	1= if the respondent reported very good mental health status, 0 otherwise
Good	1 = if the respondent reported good mental health status, 0 otherwise
Fair/Poor	1 = if the respondent reported fair or poor mental health status, 0 otherwise
Geographic region	
Halifax regional municipality	1 = if the respondent resides in Halifax, 0 otherwise
Cape Breton regional municipality	1 = if the respondent resides in Cape Breton, 0 otherwise
Other regions	1 = if the respondent resides in the cumulative other regions of Nova Scotia, 0 otherwise

Abbreviation: CIW, Canadian Index of Wellbeing.

###  Statistical Analysis 

####  Measuring Socioeconomic Inequality in Well-Being

 This study uses the C approach to measure the level of socioeconomic inequality in well-being. As a summary of the measure of socioeconomic inequality, this index is a preferred measure of socioeconomic inequality in well-being because it meets three important criteria: (1) it captures the impact of socioeconomic factors on well-being inequality, (2) it represents the entire population, and (3) it is responsive to changes in the socioeconomic distribution of the population.^45^

 The calculation of the C for a certain outcome variable, in this case, well-being, is based on the Concentration curve (CC), which displays the cumulative percentage of an outcome variable on the vertical axis against the cumulative share of the population ranked by increasing SES (as measured by income) on the horizontal axis. When the population experiences a similar level of well-being, the curve follows a 45-degree line representing perfect equality. If the CC is below (above) the 45-degree line, the value of C for well-being is positive (negative), indicating that well-being is more concentrated among the higher (lower) SES groups. The C is defined as twice the area between the CC and the 45-degree line. The *C* ranges between -1 and +1, with zero indicating perfect equality.^46^

 The C for well-being was calculated using the “convenient regression” technique as described below^47^:


(1) $$2\sigma_{R}^{2}\left(\frac{WB_{i}}{\mu }\right)=\alpha +\rho R_{i}+\varepsilon_{i}.$$

Where *WB*_i_ represents well-being for individual *i*, *μ* is the mean of the well-being variable across the entire sample. *R*_i_ = *i /N* is the fractional rank of individual *i* in the SES distribution, where *i* ranges from 1 to *N*, representing the poorest and richest individuals, respectively. σ_R_^2^ is the variance of fractional rank. The estimate of *ρ*, obtained through ordinary least squares estimation, corresponds to the C and its standard error.^48^

####  Determinant of Socioeconomic Inequality in Well-Being

 By decomposing the C, we can measure how much of the observed socioeconomic inequalities in well-being in Nova Scotia, Canada, are attributed to known factors that impact well-being, such as sociodemographic characteristics, SES, and mental health measures. Assuming a linear regression model that links our well-being variable, *WB*, to a set of *k* explanatory variables, *x*_k_, as follows:


(2) $$WB=\alpha +\sum_{k}\beta_{k}x_{k}+\varepsilon .$$

The C for *WB* can be broken down into the contribution of explanatory factors as shown below^49^:


(3) $$C=\sum_{k}\left(\frac{\beta_{k}\bar{x_{k}}}{\mu }\right)\;C_{k}+\frac{GC_{\varepsilon }}{\mu }.$$

According to equation 3, the *C* of the well-being variable is equivalent to a weighted sum of the *C* of the explanatory variables, *C*_k_, with the weight being the elasticity *WB* of concerning $x_{k}:\beta_{k}\left({\bar{x}}_{k}/\mu \right)\;with\;{\bar{x}}_{k}$ denotes the mean of *x*_k_. If an explanatory variable has a significant elasticity and an unequal SES distribution, it will contribute to the socioeconomic inequality in well-being. The *GC*_ε_ is generalized *C* for the error term defined as: $\sum_{i=1}^{n}\varepsilon_{i}R_{i}.$ ^49^ The error term component in equation 3 indicates the socioeconomic inequality in well-being that is not explained by the variation in the included explanatory variables among individuals with different levels of SES.^48^

 According to equations 3, the “contribution” of each explanatory factor (refer to as absolute contribution) to the C for well-being can be calculated as: $\left(\frac{\beta_{k}{\bar{x}}_{k}}{\mu }\right)C_{k}.$ The term “contribution” indicates the degree to which the observed association between SES and well-being in Nova Scotia, Canada, can explain the variation of an explanatory variable across different SES groups and its relationship with well-being. A positive (negative) contribution of a particular explanatory variable to the C for well-being implies that the SES distribution of that variable and its association with well-being contribute to a higher level of well-being among the high (low) SES group. To ensure that survey estimates are representative of the population in Nova Scotia, we adjusted for sampling weights in the analyses.

## Results

###  Descriptive statistics

Table 2 reports the descriptive statistics for the variables used in the study. The average age of the respondents is 50.7 years. The sample is distributed evenly between males and females. Most individuals in the sample (73%) are married or living common law, while the remaining participants are either single/never married or separated/widowed/divorced. A 15% of the sample identifies as belonging to an ethnic minority group. The average equivalized household income in the sample is approximately $62 000 per year. In terms of mental health status, only about 12% of individuals in the sample reported having an ‘excellent’ level of mental health. A large portion of the sample (81%) has an education level greater than a high school diploma. The average score for well-being measures (CIW) is 4.66, with a standard deviation of 1.11.

**Table 2:** Descriptive Statistics of Variables Used in the Study

**Variable Name**	**Mean (SD)**
**Outcome variable**	
Well-being (CIW)	4.66 (1.11)
**Independent variables**	
Sociodemographic variables	
Age	50.73 (16.44)
Male	0.49
Marital status	
Single, never married (ref.)	0.15
Married or living common-law	0.73
Separated, divorced, or widowed	0.13
Ethnicity	
Minority (ref.)	0.15
Non-minority	0.85
Socioeconomic status	
Equivalized household income (CAD$)	61 762 (44 293)
Education	
Elementary or high school (ref.)	0.19
Post-secondary, trade	0.20
College diploma	0.18
University degree	0.28
Graduate degree	0.15
Employment status	
Employed (ref.)	0.62
Unemployed	0.04
Retired	0.27
Other employment	0.07
Mental health	
Excellent	0.12
Very good	0.39
Good	0.32
Fair/Poor (ref.)	0.17
Geographic region	
Halifax regional municipality (ref.)	0.45
Cape Breton regional municipality	0.10
Other regions	0.45

Abbreviations: CIW, Canadian Index of Wellbeing; SD, standard deviation. Note: ref. indicates base category in the decomposition analysis.

###  Socioeconomic Inequality in Well-Being in Nova Scotia

**Figure 1:**
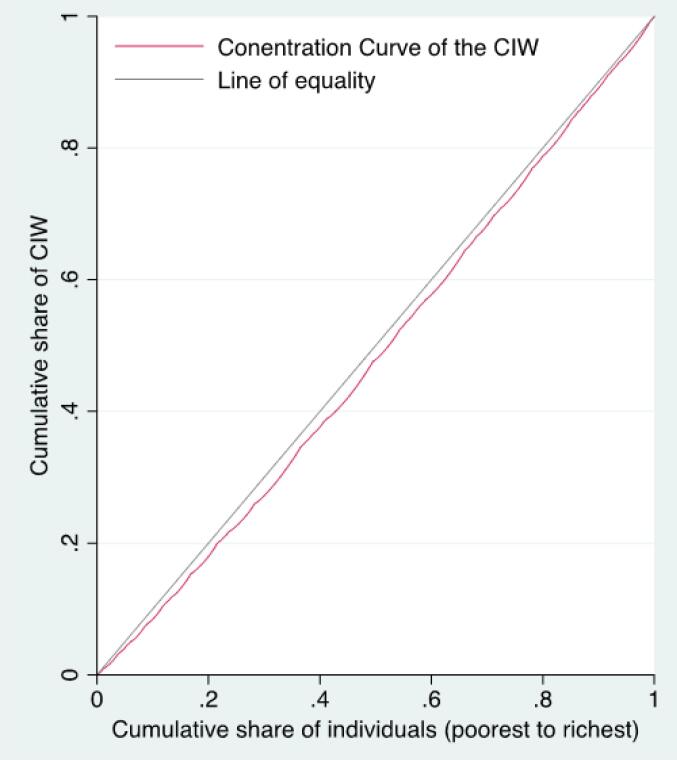
The Concentration Curve for the Canadian Index of Wellbeing in Nova Scotia, Canada. Abbreviation: CIW, Canadian Index of Wellbeing.

Figure 1 demonstrates the CC of well-being in residents in Nova Scotia. The CC lies below the 45-degree diagonal line, which represents that well-being is more concentrated among wealthier individuals in Nova Scotia. The positive value of the (C = 0.0294; 95% confidence interval: 0.0267 to 0.0321) indicated slightly higher well-being among the wealthier Nova Scotians. While the C indicates the presence of statistically significant socioeconomic inequality in well-being, it is important to explore the underlying determinants of these inequality through decomposition analysis. Understanding the specific factors contributing to these inequalities can offer valuable insights for policy-makers, even when the overall inequality measure may not appear large.

###  Decomposition of the Socioeconomic Inequality in Well-Being in Nova Scotia

Table 3 depicts the results of the decomposition of the socioeconomic inequality in well-being in Nova Scotia. Within the table it contains the estimated coefficients on explanatory variables stemming from the regression model, the elasticises, the concentration index of the explanatory variables (*C*_k_), and the contribution of each explanatory variable to the C.

**Table 3:** Decomposition of the Socioeconomic Inequality in Well-Being in Nova Scotia, Canada

**Variables**	**Coefficient**	$\bar{x}$	**Elasticities**	***C***_k_	**Contribution to ** ***C***			
					**Absolute**	**Absolute Summed**	**%**	**% Summed **
**Sociodemographic variables**								
Age	0.0075^a^	50.7265	0.0816	0.0072	0.0006		1.99	
Male	0.0430	0.4925	0.0045	0.0501	0.0002		0.77	
Marital status								
Single, never married (ref.)								
Married or living common-law	-0.0155	0.7265	-0.0024	0.0887	-0.0002		-0.73	
Separated, divorced, or widowed	-0.2064^a^	0.1283	-0.0057	-0.1962	0.0011		3.80	
Ethnicity								
Minority	0.0566	0.7401	0.0090	0.0429	0.0004	0.0021	1.31	7.15
Non-minority (ref.)								
**Socioeconomic status**								
Equivalized household income^d^	0.0023^a^	61763	0.0302	0.3717	0.0112	0.0112	38.23	38.23
Education								
Elementary or high school (ref.)								
Post-secondary, trade	-0.0717	0.1963	-0.0030	-0.1487	0.0004		1.53	
College diploma	-0.1362^b^	0.1779	-0.0052	-0.0666	0.0003		1.18	
University degree	0.0572	0.2835	0.0035	0.1485	0.0005		1.76	
Graduate degree	0.0937^b^	0.1532	0.0031	0.3779	0.0012	0.0025	3.96	8.43
Employment status								
Employed (ref.)								
Unemployed	-0.2376^b^	0.0395	-0.0020	-0.4755	0.0010		3.26	
Retired	0.4590^a^	0.2708	0.0267	-0.0733	-0.0020		-6.66	
Other employment	-0.1273^c^	0.0693	-0.0019	-0.2914	0.0006	-0.0004	1.88	-1.52
Mental health								
Excellent	1.6145^a^	0.1243	0.0430	0.1179	0.0051		17.27	
Very good	1.1832^a^	0.3865	0.0981	0.0868	0.0085		29.01	
Good	0.6658^a^	0.3232	0.0461	-0.0464	-0.0021	0.0114	-7.29	38.98
Fair/Poor (ref.)								
Geographic region								
Halifax regional municipality (ref.)								
Cape Breton regional municipality	-0.1359^b^	0.1002	-0.0029	-0.1079	0.0003		1.07	
Other regions	0.0513^a^	0.4462	0.0049	-0.1010	-0.0005	-0.0002	-1.69	-0.62
**Sum**						0.0266		90.66
**Residual**						0.0027	9.34	9.34
**Total C**						0.0294	100	100

Note: The (absolute) contribution of each variable was calculated by multiplying the elasticity of that explanatory variable by its corresponding C. The percentage of contributions were determined by dividing the (absolute) contribution by the C for well-being (ie, 0.0294), and then multiplying the result by 100. All the percentage contributions should add up to a total of 100%. a^a^*P* < .001, b^b^*P* < .05, and b^b^*P* < .0.1. ^d^To improve readability, the coefficient associated with equivalized household income was multiplied by 1000.

 The elasticity column represents the responsiveness of the well-being measure to a change in each explanatory variable. The positive sign (negative) in the elasticity indicates an increase (decrease) in well-being measure in an association with a change in the explanatory variable. According to the results presented in the table, several variables displayed positive elasticities, including age, sex (being male), income, higher educational attainment, and better mental health status. On the other hand, variables such as unemployment and marital status (being separated, divorced, or widowed and being married or living in common-law) showed negative elasticities.

 The negative (positive) value of *C*_k _for a particular variable signifies that the predictor is more prevalent among the less affluent (richer) residents. Based on the results reported in Table 3, variables such as being married or in a common-law relationship, holding a graduate degree, and having an excellent mental health status were more prevalent among the richer population. Conversely, variables like having a good mental health status or possessing a post-secondary trades certificate showed a higher concentration among the poorer residents.

 The calculated contribution of the predictors on the C indicate that all SES variables (excluding the retirement status) have demonstrated a positive contribution (ie, they amplify the concentration of CWI among the wealthier population) to the socioeconomic inequality in well-being within Nova Scotia. Conversely, in relation to sociodemographic variables, it should be noted that being married or engaged in a common law relationship has been demonstrated a negative contribution to the socioeconomic inequality in well-being.

**Figure 2:**
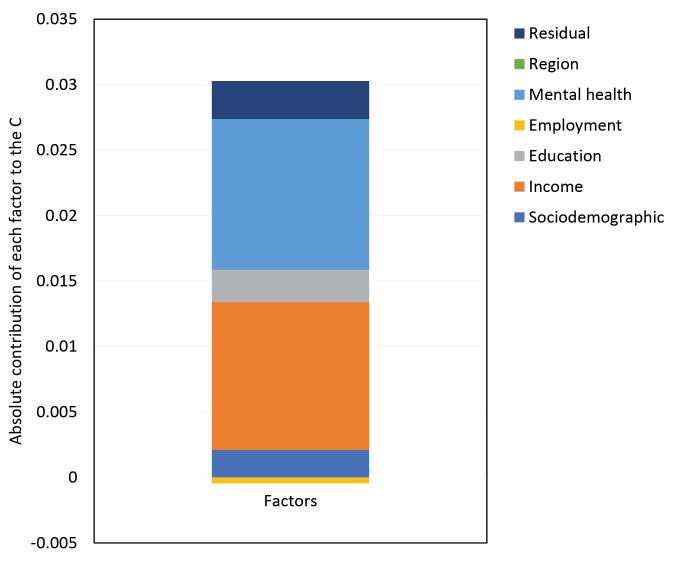
Absolute Contribution of Each Factor to Socioeconomic Inequality in Well-Being in Nova Scotia, 2019. Note: The sum of all the absolute contributions adds up to a total of C for well-being.

Figure 2 illustrates the absolute contribution of each explanatory variable on the socioeconomic inequality in well-being observed in Nova Scotia. This graphical representation, along with the numerical data detailed in Table 3, underscores that the mental health status (accounting for 38.98%), the equivalized household income (constituting 38.23%), and education levels (comprising 8.43%) stand out as the main predictors contributing to the observed socioeconomic inequality in well-being within the province of Nova Scotia.

 Moreover, the findings indicate that the explanatory variables incorporated in the model account for a substantial 90.66% of the observed socioeconomic inequality in well-being. However, it is noteworthy that an unexplained component of roughly 9.34% remains (termed as the residual component). This residual percentage indicates the potential presence of some explanatory variables not included in the current model, which nevertheless may exert some influence on the socioeconomic inequality in Nova Scotia.

## Conclusions

 Enhancing public well-being is a major policy goal, and governments employ diverse strategies to improve various aspects of individual well-being.^3,9,50,51^ Understanding societal well-being is crucial, but equally important is examining its distribution within society.^1,9,16^ A comprehensive understanding of the distribution of well-being throughout the population provides valuable information for policy-makers to take more targeted approaches in implementing certain policy initiatives to help alleviate the unequal distribution of well-being. Using a unique dataset of NSQLS and the *C* approach, we measured and decomposed socioeconomic inequality in well-being in the province of Nova Scotia, Canada.

 Our findings indicated a positive correlation between older age and well-being of Nova Scotians. This finding is consistent with prior literature on how aging is associated with greater levels of well-being.^52,53^ This result is also consistent with a more recent study focusing on well-being and older adults throughout the COVID-19 pandemic.^54^ It should be noted, however, that aging and well-being literature exhibits considerable variation in its findings regarding positive associations.^55^ In fact, there are conflicting findings regarding whether aging is associated with improved measures of well-being. Furthermore, being separated, divorced, or widowed showed a negative association with the well-being of individuals. The results also emphasized the influence of SES variables: high income and education, as well as retirement, were found to have a positive association with well-being. Income, education, and retirement were found to be associated with increases to well-being in previous work as well.^56-58^ Results from our analysis also showed a strong association between mental health and well-being of residents of Nova Scotia. The latter finding is consistent with several previous studies.^59,60^

 Our analysis reveals a positive but modest C, indicating that wealthier Nova Scotians experience slightly higher well-being. The finding is consistent with prior research.^10,14,61,62^ Considering all other possible contributing factors to the inequality in well-being of the population, income is the factor that has been most widely studied and focused on in prior research.^17,22^ Consistent findings suggest that individuals who fall into the higher income bracket levels have increased well-being measures.

 While we observed modest socioeconomic inequality in well-being, its statistical significance warrants an examination of the contributing factors through decomposition analysis to better understand the potential for targeted interventions. The decomposition of the C for well-being indicates that the highest contributing factors to socioeconomic inequalities in well-being in Nova Scotia were mental health status, income itself, and education levels, respectively. These results suggest that there is a need to address the poor mental health status amongst the financially poor to improve inequality in well-being among the population. Implementing targeted mental health initiatives aimed at the low SES segment of the population represents a crucial initial step to alleviate the burden of inequality in overall well-being.

 Our study is subject to some limitations. First, due to cross-sectional design of the study, we cannot establish temporality between determinants and socioeconomic inequality in well-being. Second, as the dataset is secondary, there are inherent limitations in having complete control over the measurement and selection of the constructs. Third, since the survey data being self-reported for many constructs, the issue of some bias and social desirability may occur. Lastly, our results include some demographic and geographic uniqueness which exist specifically in Nova Scotia. There may indeed be differences between provinces – all which may limit the applicability of our results to broader Canadian contexts. Future research could address this limitation by applying similar methodological approaches to data from other geographic areas or communities. There are ample opportunities, given data availability, to explore the determinants of socioeconomic inequalities in well-being across different provinces in Canada and globally, capturing the variability in different regions.

 Despite certain caveats, our study highlights the multifaceted challenges underlying well-being inequality in Nova Scotia, emphasizing mental health, education, and income inequalities as significant barriers. Addressing these interconnected issues requires a comprehensive approach. Given that mental health was identified as a key factor intertwined with these inequalities, targeted policies aimed at addressing mental health challenges among socioeconomically disadvantaged groups are essential for reducing well-being inequalities. This could potentially be accomplished by improving access to mental health services and raising awareness of mental health issues within low SES populations.

## Acknowledgements

 The authors thank participants of the 2023 Canadian Society for Epidemiology and Biostatistics (CSEB) Biennial National Conference for their comments and suggestions.

## Ethical issues

 The Dalhousie University’s ethical review board granted approval for this study (REB #2022-6277).

## Conflicts of interest

 Authors declare that they have no conflicts of interest.

## Availability of data and material

 The data sets utilized in this study are accessible from the Enage Nova Sctia for the approved research project.

## Code avaibility

 The code can be obtained from the author upon a reasonable request.
